# Heavy metal contamination from textile wastewater and its health impacts: a case study from West Bengal with sustainable remediation approaches

**DOI:** 10.1038/s41598-025-13357-w

**Published:** 2025-08-12

**Authors:** Biman Gati Gupta, Rishika Mukhopadhyay

**Affiliations:** Elitte College of Engineering, Maulana Abdul Kalam Azad University of Technology, Kolkata, India

**Keywords:** Metals, Fruits and vegetables, Impact, GI disorder, Disease profile, Remediation, Eco-planning, Nanoparticle., Biotechnology, Ecology, Neuroscience, Climate sciences, Ecology, Environmental sciences, Environmental social sciences, Diseases, Gastroenterology, Risk factors, Chemistry, Engineering, Nanoscience and technology

## Abstract

The presence of heavy metals in the environment is a common risk factor for a number of gastrointestinal (GI) disorders worldwide. We oversaw an inquiry in the Calcutta textile industry area from 2022 to 2023, which has been engaged in textile production for over a century. We investigated the impact of heavy metals (Pb, Cd, and Zn) on soil, fruit, and other agro-samples as well as their toxicity in the waste of small-scale BD units. “The investigation was sparked by the gastrointestinal illnesses that the local population was suffering. In that area and its hinterland, people utilize surface water and eat everyday agro-products that are 50 times less rich in zinc than predicted and contain 2 to 40 times more Pb, Cd, and Ni than the WHO/FAO (2022) standard limit. The region’s soil tested positive for potentially harmful metals, indicating that there may be an epidemiological risk of cancer, ulcers, and gastrointestinal ailments. This finding was also verified by statistical analysis and 2 years of observations/surveys with residents. The remediation processes save public health, millions of liter of groundwater and the ecology of the earth at large.

**Supplementary Information:**

The online version contains supplementary material available at 10.1038/s41598-025-13357-w.

## Introduction

**Fig. 1:**
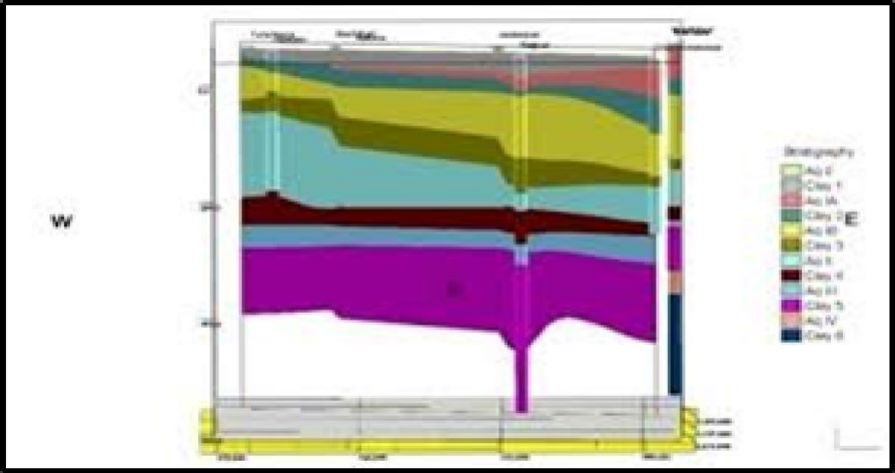
Groundwater map in South 24 Parganas, West Bengal.

As per the study in an area of the Bengal basin (latitude 21°45′N to 22°30′N, longitude 88°15′E to 88°30′E) located in the southern side of the Indo-Gangetic Plain, which is also the largest alluvial tract in the world. The district of South 24 Parganas, southern part of Kolkata, West Bengal India has largely saline water originating mainly in the upper to intermediate aquifer while “sweet” water is situated in limited lower aquifers. The growing textile bleaching and dyeing (BD) industries and the population in this area have increased the water demand from groundwater resources for drinking, agriculture, and industry. The Central Ground Water Board, India endorsed that between 2017 and 21 the groundwater table in Kolkata has dropped by 2.1 m which is a drop of 18.6% over the level documented five years ago. For South 24 Parganas, (Site) the drop-in ground water table is 2.5 m or 27.8% from what it was five years ago (Fig. 1.)

Estimates indicate that an average of 70–150 L of water, 0.6 kilogrammes of sodium chloride, 40 grammes of reactive dyes (both organic and inorganic), alkalis (sodium hydroxide), and dyeing auxiliaries are required to dye 1 kilogramme of cotton products using reactive colours^1^. Wastewater from textile bleaching and dyeing (TDW) is consistently discharged into the environment, modifying the biological, chemical, and physical properties of natural resources and endangering global sustainable biodiversity^2,3^.

In this situation, water was extracted from subsurface sources consuming BD units. Significant quantities of untreated effluent from these sectors are discharged into the environment. Throughout several phases of the process, wastewater generated an array of metals and chemicals^4^. Wastewater and untreated sewage contain elements such as cadmium, chromium, nickel, arsenic, and lead, which ultimately permeate soil, agricultural goods, and subsequently enter human bodies via the food chain. The accumulation of hazardous metals adversely affects agricultural output. The metals lead (Pb), nickel (Ni), cadmium (Cd), arsenic (As), and chromium (Cr) are hazardous. They influence the yield and quality of surface water, soil, and agricultural products such as crops, fruits, and vegetables^5^.The capacity of agricultural products to sequester hazardous metals from polluted soil and water is crucial for aquatic organisms, humans, and domestic animals^6,7^. The ingestion of concentrated metals in fruits, vegetables, and leaves presents health hazards^8^.

The Central Pollution Control Board (CPCB) of India reports that 80% of hazardous trash, including heavy metal contamination, originates from the states of Gujarat, Maharashtra, and Andhra Pradesh^9^. Numerous textile industries are located in Solapur, in the Indian state of Maharashtra. Accessed on May 26, 2022. Department of Gazetteers. SOLAPUR, 2006. Research has shown that the textile sector contributes to heavy metal pollution^10^. 90% of the metals ingested by people are derived from fruits and vegetables, while the remaining 10% is acquired through dermal contact and inhalation of contaminated dust^11–14^. Consequently, quantifying the concentration of heavy metals in frequently consumed fruits and vegetables is essential to assess the potential danger to human health^15,16^. Previous study has primarily focused on a limited variety of vegetable kinds^8,17^. To our knowledge, no studies have recorded the accumulation of heavy metals in fruits and vegetables from the Solapur region. Moreover, in contrast to developed nations, developing countries like China, France, Italy, and Morocco experience more acute public health challenges and heavy metal toxicity^18–20^. Consequently, it is imperative to assess the risk of heavy metals to human health through the analysis of commonly consumed fruits and vegetables.

Heavy metals accumulate in the and fatty tissues human skeleton, important to reduction of key nutrients and resulting in central nervous system shortages, in addition to cardiac, gastric, hepatocellular, renal, neurodevelopmental, reproductive, and immune disorders, as well as intrauterine retardation^21^.

As stated in^22^, the proliferation of this detrimental unit is associated with various ecological risk factors, including the accessibility of human resources possessing abilities in sewing, knitting, and other competencies necessary for the production and repair of garments adversely affected by the poor socioeconomic conditions in these regions. This study aimed to investigate the concentrations of toxic metals in various water sources, including raw wastewater, surface water, agricultural land, and its products, while also assessing the potential health risks to humans from food consumption and contact with contaminated water, along with strategies for risk mitigations.

### Significant of current study

Aims to asses : Toxic and Heavy Metals Contamination, Environmental and Human Health Risks, Sources of Contamination, Impact on Ecosystems, Regulatory Standards (EU, WHO, FAO), Recommendations for Mitigation, Scientific and Policy Implications, Analytical Methods, and Global Relevance.

### Global relevance

Broader implications for urban and industrial areas worldwide facing similar challenges in wastewater management and environmental contamination both in South Asian Countries like India, Pakistan, Thailand, South Korea etc. and developed countries of Morocco, France and Italy etc.

## Materials and methods

### Study area

**Fig. 2:**
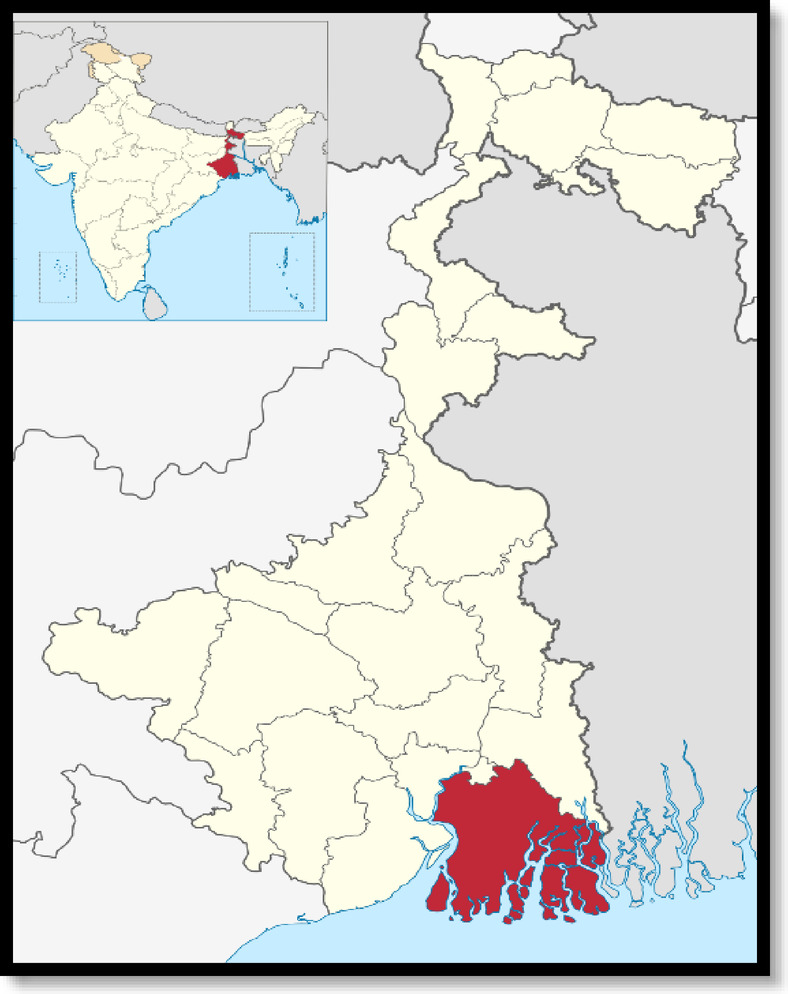
Map of India & West Bengal and South 24 Parganas. (Source: Wikimedia Commons)

Alipore Sadar subdivision is the most urbanized part of the South 24 Parganas district. 59.85% of the population lives in the urban areas and 40.15% lives in the rural areas. In the northern portion of the subdivision there are 21 census towns. The entire district is situated in the Ganges Delta and the subdivision, on the east bank of the Hooghly River, is an alluvial stretch, with industrial development. Location of core area Maheshtala is located at 22°30′22″N 88°15′01″E (Fig. 2). It has an average elevation of 7 m (23 ft). Temperatures of the area varies from 25 °C in December (winter) to 35 °C in May (summer).

**Fig. 3:**
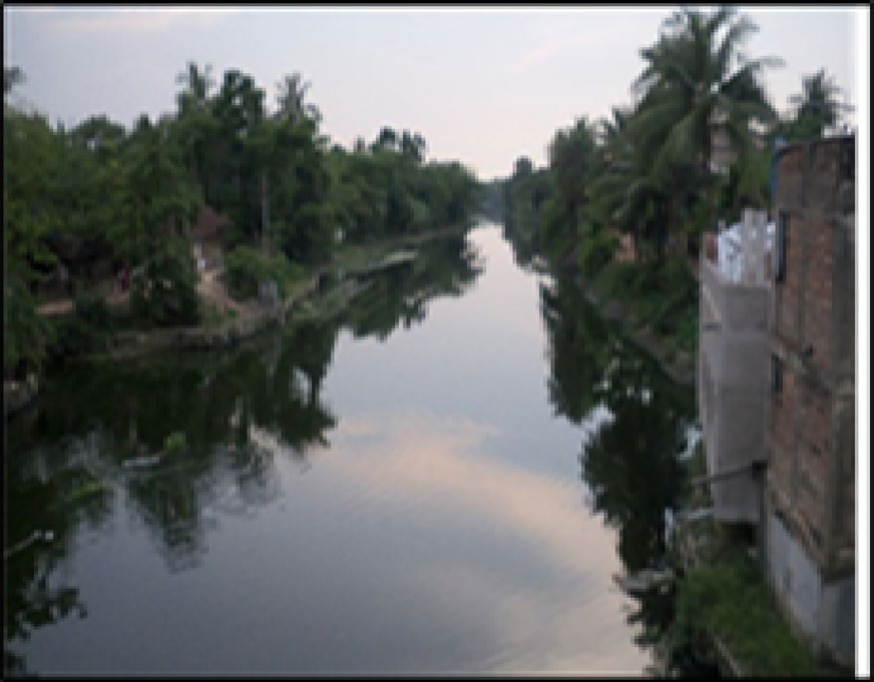
Contaminated canal.

Rameswarpur, Chata Kalikapur, Ganye Gangadharpur and Asuti form a Textile cluster of census towns on the southern side of Maheshtala, as per the map of the Thakurpukur Maheshtala for the South 24 Parganas district. is the major sources of Chemical and metal (from wastewater) contamination of surface water, soil, fruits and vegetable. Due to food- chain action local residents are suffering from metal contained food. Climate is Köppen-Geiger climate classification system classifies its climate as tropical wet and dry (Aw).The current investigation was carried out throughout the summer, rainy, autumn and winter (2022–23). A total number of 31 effluent samples were collected, and 4 samples were taken from different locations in the area around the existing canal (Fig. 3). In both years, the samples were taken at different times of the year. All of the samples were subjected to physicochemical testing as part of our study.

### Determination of heavy metals in water and soil

The present study aimed to determine the composition of toxic metals (Cr, Mn, Cu, As) and heavy metals (Cd, Ba, Hg, Pb) in soil and water by an inductively coupled plasma optical emission spectrometer (ICP-OES). To ensure accuracy during the analysis of Cr, Mn, Cu, As, Cd, Ba, Hg, and Pb in real samples, certified reference material CRM, SRM 2709a of San Joaquin soil and water SRM 1640a were analysed and results were presented.

### Collection and preparation of fruits and vegetable samples

Randomly 32 fruits and vegetable samples were collected from local market. The fruits and vegetable samples were air-dried at 35 °C for 72 h using an air circulation oven (Vision Scientific, Korea), crushed, passed through a sieve of mesh size No. 10, and stored in polyethylene bottles before acid digestion followed by metal analysis. 1 g of soil representative sample was weighed and transferred to porcelain crucible already contained 10 mL mixture of HNO_3_ and HF (1:1), the residue was formed after drying in a water bath after which the residue was dissolved in 20 mL HNO_3_ (2 M). Whatman No. 41 filter paper was used to remove the particles in the resulting solution, then the solution was transferred to a 100 mL measuring flask and made the volume up to mark with ultrapure water, ( 24). Wastewater, surface water, and subterranean water samples were collected, preserved, tested, and observed by the APHA (2020) standard, whereas agricultural products were subjected to the FAO (2017) standard.

### Water quality index: (WQI)

Based on the physic-chemical parameters of the surface water of the area as follows:

- Temperature = 29 °C, TSS = 36 mg/L, DO = 1, BOD = 104 mg/L and Conductivity = 400µs/cm.
- As per Perdue University, USA, the Water Quality index = 34 (Fair water quality).
- The index ranges from 1 to 100; a higher number indicates better water quality.

The following indexes are shown the quality of surface water:

- SRDD Index (1970) WQI(25–40)=indicates Bad water.
- Bascaron Index (1979) WQI(25–50)=indicated Bad water.
- NSF index (1965) WQI (25–40)=indicates Bad Water.

### Methods

#### Human health risk assessment

The human health risk assessment of the people in the cluster was studied both on the basis of consuming daily contaminated fruits and vegetable, using surface water for daily household work. Subsequently observed effect of metal on the health through oral questionnaire based survey of the local residents. Before initiating the research, questionnaire have been prepared with the help of IISWBM, a more than 75 years old renowned Kolkata based oldest management institute under Calcutta University, India for oral survey. This is to state that all the methods were carried out by relevant guidelines and regulations. This is also confirmed that informed consent was obtained from all subjects’ e.g. socio-economic, health etc. from local residents.

The oral based research was approved by the ethics committees of two Indian universities: HOD, Ecological Studies at Kalyani University (KU), and H.O.D., Environmental Management at IISWBM, Calcutta University (CU) to ensemble necessary ethical clearances. To protect the respondents’ anonymity corresponding author, KU and an MBA of IISWBM, CU, carried out the verbal field observation via a format developed and approved by the ethics committee. All procedures were executed in strict adherence to all applicable rules and regulations^25^. Furthermore, all plant-related studies and experiments have adhered to all applicable local, state, federal, and international regulations, including those about fruits and vegetables (such as papaya, guava, etc.) and cultivated or wild plant materials.

##  Results

### Sample analysis for heavy metals

#### Instruments

*Spectrophotometer* for use at 510 nm, providing a light path of 1 cm or longer. pH meter Separator funnels: 250mL Squibb type. Clean all glassware, including sample bottles, with 1 + 1 HNO_3_. Rinse thoroughly with distilled water. Automatic dispensing burettes: Used for all reagents to minimise indeterminate Contamination errors.

A Shimadzu Atomic Absorption Spectrophotometer 6300 model with air-acetylene flame of an average fuel flow rate between 0.8 and 4.0 L/min, and the support gas flow rate between 13.5 and 17.5 L/min was used. The single element hollow cathode lamps used in AAS were of Hamamatsu Photonics Co. Ltd., L2433 series. The standard references for the given elements were procured from Inorganic Ventures Inc. and Sisco Research Laboratories Mumbai Ltd. Calibration curves for various elements obtained from these standards were of first order reaction^26^ .

The allowed levels of hazardous metals, including Pb, Ni, Zn, and Cd, in soil and other agricultural products are listed in Table 1 by the World Health Organization and the Food and Agriculture Organization of the United Nations in 2011^26^. Wastewater, surface water, deep tube wells, and farmland are estimated to have elevated concentrations of the harmful elements Pb, Ni, Zn, and Cd, according to Tables 2, 3 and 4, and 5. The kinds of metals in many fruits and vegetables are shown in Table 6. We were able to collect 31 samples in two years. The samples comprised 36 items, including canal water (9), raw effluent (8), surface water (5), deep-tube water (2), and soil (6), and 6 obtained Nd = not detectable.

**Table 1:** Safe permissible limit of heavy metals in fruits, vegetables, water, and soil as FAO/WHO standard 2020, and IS: 10,500: 2017^30,31^.

Metal	Fruits/veg. (mg/kg)	Soil (mg/kg)	Water (mg/l)	Papaya (mg/kg)	Guava (mg/kg)	Effluent IS:10,500 (mg/l)
Cu	73	5-5.6	2			3.0
Pb	0.30	2-13.4	0.01	Nd	0.58	0.10
Cd	0.20	0.1	0.003	Nd	Nd	1.0
Cr	0.1-1	10–80	0.05			2.0
Zn	99.4	60–780	3			15
Ni	1–10	10–50	0.02	0.26	Nd	3

Nd = not detectable, Source a = Adue et al. 2012.

**Table 2:** Pb concentration in effluent, canal, pond, tube well water and soil.

Sl. no	Sample & year 2022-23	Canal water (mg/l)	Effluent (mg/l)	Pond water (mg/l)	Tube well water (mg/l)	Soil (mg/kg)
1.	S-1	0.05	0.07	0.01	0.007	1.16
2	S-2	0.05	0.08	0.25	–	11.14
3.	S-3	0.104	–	–	–	17.32
4.	S-4	0.03	–	–	–	41.20
5.	S- 5	0.014	0.25	0.016	0.058	90.80
6.	S-6	0.15	1.84	0.01	–	–
7.	S-7	0.10	0.10	–	–	–
8.	S-8	0.10	0.14	–	–	–
9.	S-9	**–**	0.17	–	–	–
	Mean	0.38	0.074	0.07	0.02	32.32
	S.D	0.27	0.67	0.10	0.02	32.07

S-1-4, S:5–9 indicate samples of the 1st year and 2nd year. As per WHO safe limits of Pb concentration in canal water, Effluent, Pond water, tube well water, and soil were very significantly higher (39-fold, 7-fold, 7-fold, 10-fold). Further, the Mean Pb concentration was found in canal water > soil > effluent > Pond ≥ tube well water. Again, Pb levels increased from the 1st year to the 2nd year in canal water (0.03 mg/l to 0.15 mg/l), effluent (0.07 mg/l to 1.84 mg/l), tube well water (0.007 mg/l to 0.058 mg/l), and soil (from 1.16 mg/kg to 90.80 mg/kg).

**Table 3:** Content of nickel in the effluent, Canal water, pond, tube well water, and soil.

Sample/year	Canal water (mg/l)	Effluent (mg/l)	Pond (mg/l)	Tube well (mg/kg)	Soil (mg/kg)
S-1	0.05	–	–	–	0.34
S-2	0.05	–	–	–	14.93
S-3	0.07	–	–	–	34.77
S-4	0.05	–	–	–	–
S-5	0.037	0.049	0.02	0.016	–
S-6	0.037	0.093	–	–	–
Mean	0.05	0.07	0.02	0.016	16.68
SD	0.01	0.02	–	–	14.11

As per the sample in the 1st year (S: 1–4) and 2nd year (S: 5–6), mean Ni in effluent, canal, pond water, and soil were significantly higher (4-fold, 3-fold, normal, 2-fold) and lower in tube well waste.

**Table 4:** Content of zinc in the effluent, canal water, pond, tube well water, and soil.

Sample	Canal water	Effluent	Pond water	Tube well water	Soil
	Mg/l	Mg/l	Mg/l	Mg/l	Mg/kg
S-1	0.05	0.94	0.004	1.15	250.84
S-2	0.28	0.15	–	–	54.26
S-3	0.02	–	–	–	980.4
S-4	0.18	–	–	–	–
S-5	0.15	0.55	0.05	0	–
S-6	–	0.07	–	–	–
S-7	–	0.09	–	–	–
Mean	0.14	0.36	0.027	1.15	428.50
S.D	0.09	0.33	0.025	–	398.41

As per WHO limits, the mean Zn level in effluent, canal, pond, tube well water, and soil.

Very significantly lower (8- fold, 21-fold, 111- fold, 3- fold, 2- fold).

**Table 5:** Content of cadmium (Cd) in soil.

Sample/year	Soil mg/kg
S-1	0.94
S-2	0.53
R-3	1.29
Mean	0.92
S.D	0.31

As per WHO standards, the concentration of Cd in soil is lower (9 fold).

**Table 6:** Metal content in different fruits and vegetables.

Metal	Papaya mg/kg	Coconut water mg/l	Guava mg/kg	Coix grass mg/kg	Water Hyacinth mg/kg
Pb	0.45	0.41	12.62	0.66	2.75
Cd	–	–	1.47	0.10	0.15
Cr	–	–	0.10	0.145	0.27
Zn	1.96	0.39	–	13.15	

Pb levels are significantly higher (2-fold, 1.5-fold, 42-fold, 2-fold, 9-fold), safe limits of Cd in Guava were significantly higher (7-fold), the Safe limit of Cr in Guava was normal, and the Safe limit of Zn in Papaya, Coconut water, and long grass were significantly Lower, 50-fold, 8–fold, and 8 fold, respectively.

In case of Pb, there was a 39-fold increase in canal water, a 7-fold increase in effluent, a 7-fold increase in tube well water, and a 10-fold increase in soil. Pb concentrations compared with the acceptable levels set by the World Health Organization. In addition, the sequence of decreasing mean Pb content was as follows: canal water, soil, effluent, pond, and finally, tube well water. Canal water (0.03 mg/L to 0.15 mg/L), effluent (0.07 mg/L to 1.84 mg/L), well water (0.007 mg/L to 0.058 mg/L), and soil (from 1.16 mg/kg to 90.80 mg) increased the Pb content.

The mean Ni content in the soil, canal, pond, and effluent was two times higher than that in the tube well water, threefold higher, and normal, respectively, according to the samples.

The World Health Organization reported that the concentration of Cd in soil was nine times lower than that in produce. Substantially higher concentrations of lead were detected in canal water, effluent, pond water, tube well water, and soil (38.9x, 6.7x, 7.1x, 2.1x, and 16.2x, respectively). Overall, the Pb concentrations in canal water were greater than those in soil, which in turn were greater than those in wastewater, ponds, and deep-tube well water.

The lead content in the canal water increased between 0.105 and 0.151 mg/L, whereas in the effluent, it increased from 0.08 to 0.17 mg/L. The lead content of the tube well water increased from 0.007 mg/L to 0.058 mg/L over the first two years. In the pond and deep-tube well water, the Ni content was 4.1 times greater than usual; in the surface water, it was 3.1 times greater; and in the agricultural field, it was 19 times greater. The zinc levels in the soil, canal, pond, and tube well water were far lower than what the World Health Organization considered acceptable. The Cd content in the soil sample was less than nine times. Consequently, the following is a scale for the various soil heavy metal concentrations: Lead is less abundant than nickel, zinc, and cadmium.

Papaya, guava, coconut water, long grass, and water hyacinth all presented lead (Pb) levels that were too high, according to the World Health Organization. The amount of Cd found in guava was far higher than expected. While guava’s Cr levels were within the usual range, long grass, papaya, and coconut water had zinc levels that were much lower than the FAO/WHO guidelines.

**Fig. 4:**
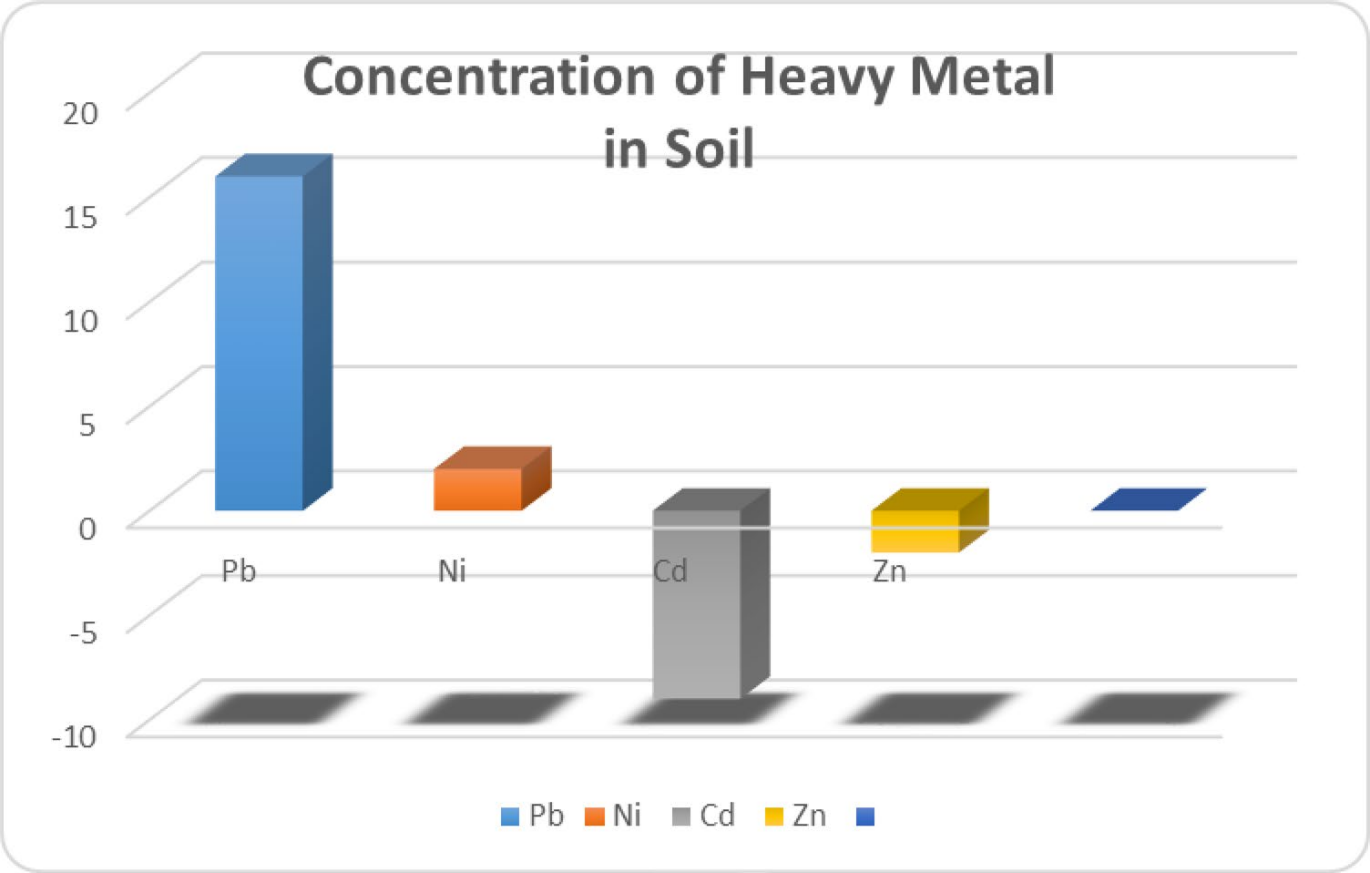
The concentration of different heavy metals in soils collected from agricultural land in the vicinity of the bleaching and dyeing units. Respective concentrations have been represented in proportionate to increased or decreased levels expressed in folds.

**Fig. 5:**
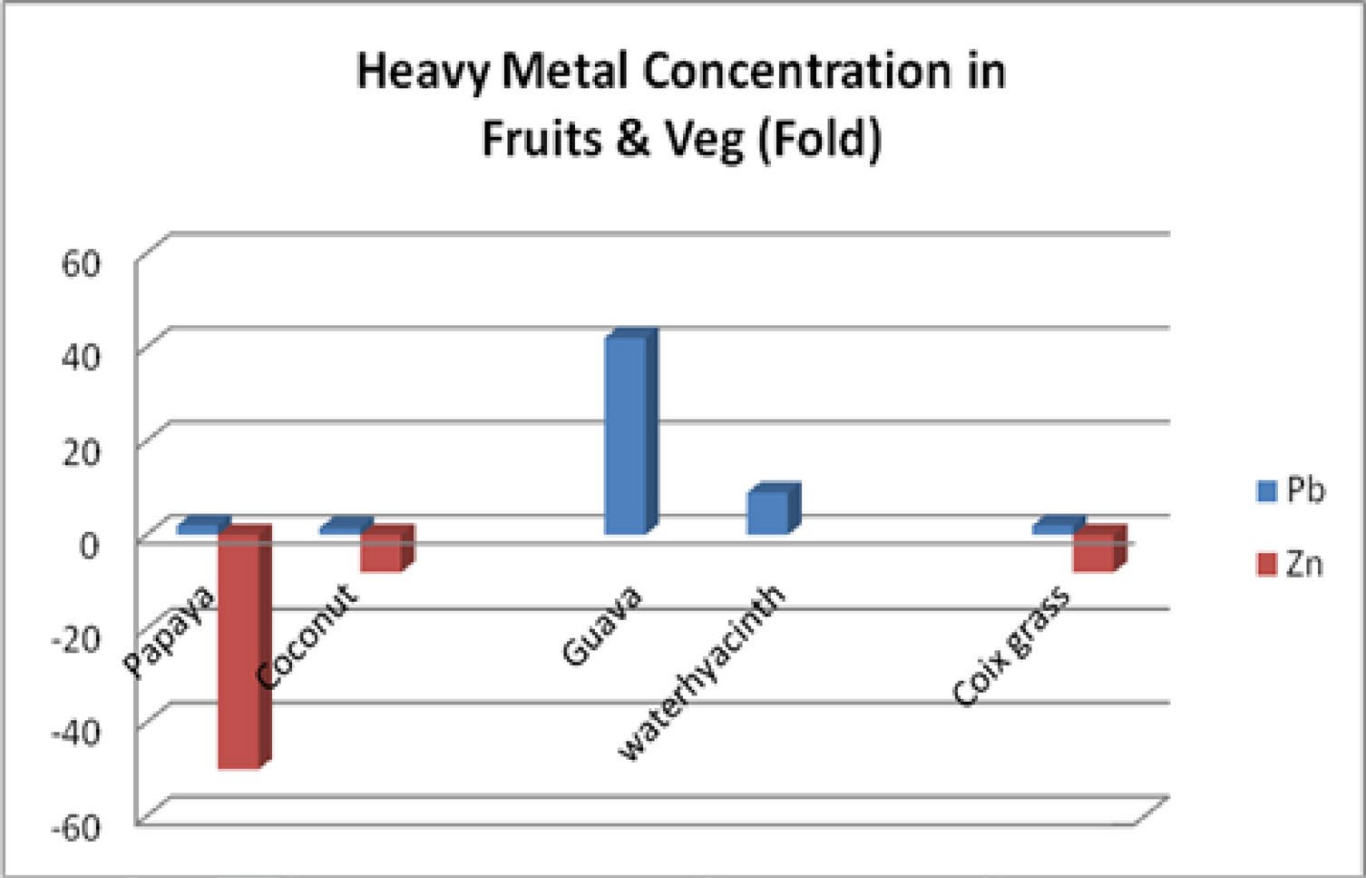
Pb and Zn concentrations in fruits, vegetables, and plants are shown either in higher or lower order (fold), respectively, against WHO/FAO and IS:10500:2020 standards.

The standard health hazard assessment results are shown in Table 8. Table 10 shows the amounts of several metals in coconut water, whereas Table 9 shows the values in guava (in milligrams per liter). Table 11 shows the disease profile based on survey findings related to heavy metal consumption, and Table 12 shows the socioeconomic condition based on survey results. Toxic metal contamination in food crops and how they contaminate them are shown in Fig. 4. The soil concentrations of certain heavy metals were emphasized. The amounts of lead and zinc in plants, fruits, and vegetables were compared to the safe limits set by the WHO/FAO and IS: 10,500: 2020, as shown in Fig. 5. The Tables and Figures section contains all of the tables and figures.

$$\begin{gathered} {\text{Hazard}}\;{\text{quotient}}\;({\text{HQ}})\;{\text{HQ}}={\text{DI}} \times \left( {\frac{{{\text{C}}\;{\text{m}}\;{\text{veg}}}}{{{\text{R}}\;{\text{f}}\;{\text{D}} \times {\text{BW}}}}} \right) \hfill \\ {\text{HI}}=\sum {{\text{HQ}}} \hfill \\ \end{gathered}$$

where DI is the daily intake of vegetable (kg day^−1^), C_M veg_ is the concentration of metal in vegetables (mg kg^−1^), BW is the body mass (kg bm) (60 kg av.), and R f D_o_ is the oral reference dose for the metals (mg kg^−1^ day^−1^). Similar to IPI, the Hazard Index (HI) which is the sum of the HQs for all the contaminants and is used to assess the overall potential for non-carcinogenic effects posed by more than one contaminant at site, HI=∑ HQ.

##  Statistical study

A one-way analysis of variance (ANOVA) and Duncan’s manifold range test were used to examine the statistical data at the 5% level in the SPSS software (version 11).


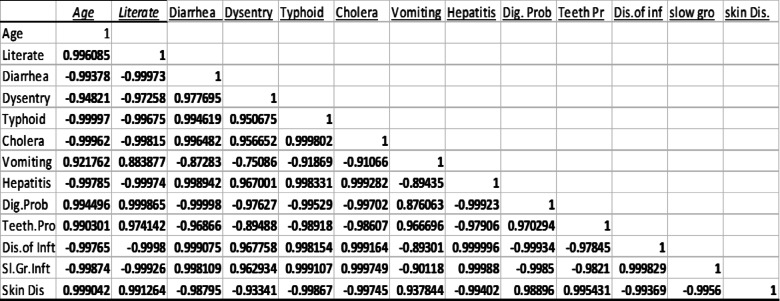


The results of the health survey and the statistical analysis revealed a substantial positive association between dysentery and diarrhea (*r* = 0.97, n ˂0.05) and a strong negative correlation between dysentery and age (*r*=-0.94, *n* ~ 0.05). Dysentery (*r* = 0.99, n˂0.05) and diarrhea (*r* = 0.99, n˂0.05) are strongly and significantly correlated with typhoid. Dysentery (*r* = 0.95), typhoid (*r* = 0.99), and diarrhea (*r* = 0.99) are strongly and significantly correlated with cholera at the 5% level of significance. At the 5% level of statistical significance, hepatitis is strongly and positively correlated with typhoid (*r* = 0.99), cholera (*r* = 0.99), dysentery (*r* = 0.96), and diarrhea (*r* = 0.99). Typhoid and digestive problems are strongly correlated negatively (*r*=-0.99, n˂0.05) and significantly correlated with vomiting (*r* = 0.99, n˂0.05). Just like tooth discoloration, gastrointestinal problems are strongly and significantly correlated with discoloration (*r* = 0.97, n˂0.05). Additionally, there is a strong negative link between skin disease and hepatitis (*r*=-0.96, n˂0.05) and a substantial positive correlation between skin disease and digestive problems (*r* = 0.99) at the 5% level. Statistical study revealed a strong association between the cluster’s water quality being negatively affected by bleaching and dyeing processes. Because surface streams are often used as drinking water sources or linked to shallow wells, chemical contamination of this water might pose health problems. Along with their obvious recreational value, streams also serve vital purposes in the food and water industries, as well as in fishing and aquaculture. Some estimates put the number of persons killed each day at around 14,000, with water contamination being the leading cause of illness and death globally. Approximately 580 individuals in India succumb to illnesses linked to water contamination daily^30^.

## Discussion

While Zn concentrations are modest, this study revealed that soils and plants absorbed substantial amounts of carcinogenic metals (Pb, Ni, and Cd). The agro-products mentioned previously are consumed regularly by the people in the core research area. On the basis of socioeconomic study conducted in this region, these individuals are classified into groups with low incomes and education levels. The domestic animals also consumed water hyacinths and tall grass. The inorganic form of harmful metals produced by everyday food, fruits, water, and air inhalation poses the greatest danger to locals, especially those with insufficient zinc vitamin intake. According to^31^ and INECAR, the study region was found to have a number of health problems, including malfunctions in the kidneys, joints, and reproductive system; neurological diseases; significant injury to the gastrointestinal tract; ulcers; and cancer.

On the basis of these results, the most prevalent places to find lead units were in canal Pb water, soil Pb, effluent Pb, pond Pb, and tube well Pb. The level of Pb in the soil is 18 times greater in the canal, at 90.80 mg/kg, as per World Health Organization regulations. Industries associated with BD discharge wastewater into adjacent canals and farmland. The lead concentrations of raw effluent were 18 times higher than the permissible limit of 0.1 mg/L established by IS: 10,500 − 2020^32^.

The calculated Ni units in the canal water tended to increase from 0.05 mg/l to 0.07 mg/l. The soil Ni concentration rose from zero to thirty-four milligrams per kilogram during the course of two years, reaching a maximum of thirty-four and a half milligrams per kilogram.

The soil Ni concentration was 3 mg/kg, which was 12 times higher than the maximum concentration specified in IS:10,500 − 2020. Canal water has zinc concentrations ranging from 0.02 to 0.18 mg/L, which is lower than the 15.0 mg/L discharge limit set by IS: 10,500: 2020. The soil Zn concentrations ranged from 54.26 to 948.4 mg/kg, with an average of 428.5 mg/kg. This value is less than the 760 mg/kg recommended by the World Health Organization. Fruits and vegetables contain low quantities of Zn. Soil, fruit, and vegetable heavy metal concentrations are shown in Tables 2, 3 and 4, and 5. Table 6 shows that zinc levels are decreased in coconut water, guava, and papaya, while the levels of lead and nickel are increased.

In the lead industry, which includes tanneries, BD units, textile mills, smelters, metal industries, and mines, workers in both developed and developing nations have been exposed to the poison. Lead is leached into the ground and water supplies via industrial effluent, and the air may be inhaled by people at some point. Abdominal discomfort, gastrointestinal disorders, symptoms of neurological breakdown, and trouble focusing are more common in children.

In 1987, the International Agency for Research on Cancer (IARC) classified lead as a probable human carcinogen. We know that lead may cause cancer and that it can have side effects, including glioma, stomach, and lung cancers.

Soil produce and horticulture produce have significant quantities of lead, nickel, and cadmium, but low concentrations of zinc. The agro-products mentioned previously are often consumed by the people in the study area. A socioeconomic analysis conducted in this area indicates that they are associated with lower socioeconomic status and lower levels of education. A large amount of water hyacinth and grass was given to the domestic animals. The most common way that people might absorb harmful amounts of metals is via food, drink, and air. It is clear that people are not getting enough zinc,” says^33^. A variety of health problems, including malfunctions in the kidneys, joints, and reproductive system; neurological sickness; and serious damage to the gastrointestinal tract, have been documented in the study region. Cancer and ulcers were also mentioned as local health issues^34^.

###  Management of toxic metals in the soil-crop system

The safety of food is essential for the well-being of all individuals. Nevertheless, toxic metals from wastewater irrigation, sludge application, and industrial effluents pose a concern. Since anthropogenic sources of hazardous metals are increasing, removing them from soil might prevent the build up of carcinogenic metals in the soil-plant system. The process by which harmful metals migrate from the soil to crops is well- understood. To keep metals from leaching into crops, it is important to lower the soil concentrations of these elements”. Equipment for cleanup must be quick, inexpensive, and environmentally friendly. Heavy metal remediation in soil may be accomplished by chemical, physical, biological, and ecological means (Fig. 7). The remediation of metal pollution might be aided by advancements in nanotechnology. The H-G approach evaluates geographical indicators, quickly identifies problematic soil areas, and builds effective remediation procedures by combining human health risk assessment with geographic knowledge. Food safety is critical for the health of the planet’s population.

## Health risk analysis

### Metals in papaya

According to the sequence of metallic concentrations in the soil where the papaya was produced, the average concentrations of various metals (mg/kg) in the papaya samples varied as follows: Na > Fe > Al > Zn > Pb (*P* < 0.05). The concentrations of three metals present in the papaya were determined lead (0.012), iron (5.1), and zinc (0.012). The vegetables did not contain any Cr or Cd, but the average concentrations of Pb, Fe, and Al were five-, one-, and one and a half-fold greater than the maximum allowable limits of metals in vegetables for human consumption, as set out by the FAO/WHO (2020) (Table 7). The U.S. Environmental Protection Agency (2002) determined that the daily consumption of papaya by adults (25–55 years old) and children (13–15 years old) significantly exceeds the threshold of 1.00 for lead (Pb), with estimated health risk quotients for adults of (36 ) of 8.78 and for children of 7.28, respectively (Table 8). Studies conducted provide further support for these findings.

**Table 7:** Concentrations of different metals in green papaya.

Parameters	Na	Pb	Fe	Zn	Al
Samples	mg/kg	mg/kg	mg/kg	mg/kg	mg/kg
S1	65.88	0.51	7.06	1.64	5.1
S2	66.72	0.62	10.86	3.01	7.56
S3	64.13	0.27	9.75	2.46	3.85
S4	72.91	0.69	6.97	3.01	8.13
S5	65.86	0.3	7.59	1.66	7.84
S6	63.46	0.75	11.57	2.2	3.88
S7	62.11	0.45	3.25	1.96	7.56
Mean	65.87	0.51	8.15	2.28	6.27
Maximum	72.91	0.75	11.57	3.01	8.13
Minimum	63.46	0.27	3.25	1.64	3.85
Std.Dev	3.49	0.19	2.84	0.58	1.92
FAO/WHO (2020)	–	0.10	5.0	3.0	6.00
Condition/fold higher	–	5	1.6	Low	1.05

**Table 8:** Health risk (HRI) assessment due to metals in papaya.

	Unit mg/kg	HQ adult	HQ child	DDI	DIM	HRI
Mean Pb concentration	0.51	8.70	7.28	0.0008	0. 003	0.86
Mean Al concentration	5.1	4.60	3.21	0.0001	0.001	0.21
Total HRI						1.07
HQ standard		1.00	1.00			1.00

DDI = Daily dietary Index, DIM = Daily intake of metal, HRI = Health risk index, Adult = 55 year.

Based on US EPA (2020) and Hakanson (1980) for HQ and US EPA (2020) for HRI.

### Metals in guava

According to Table 9, which displays the average amounts of several metals in mg/kg in the guava samples, the following metal concentrations varied significantly. The order of metallic concentrations in the soil where guava was cultivated was compatible with the order of Na > Al, Fe > Zn > Pb (*P* < 0.05), which is comparable to that of papaya.

**Table 9:** Concentrations (mg/L) of different metals in guava.

Samples	Unit	Cd	Pb	Zn
S1	mg/L	0.005	0.41	0.36
S2	mg/L	0.017	0.052	0.39
S3	mg/L	0.009	0.161	0.775
S4	mg/L	0.019	0.172	0.835
S5	mg/L	0.009	0.181	0.431
S6	mg/L	0.019	0.18	0.853
S7	mg/L	0.009	0.197	0.566
Mean		0.01	0.19	0.6
Maximum		0.02	0.41	0.85
Minimum		0.01	0.52	0.85
Std. dev.		0.01	0.11	0.22
FAO/WHO (2020)		0.003	0.01	5.0

It is evident from the mean concentrations of various metals that guava has much larger amounts of Pb (4-fold) and Al (1.15-fold). The FAO/WHO (2022) set the maximum permissible concentration. Metallic transfer and concentration were highest for Fe (3.78), followed by Zn (0.017) and Pb (0.009), according to the concentration factors of various metals in guava. In addition, compared to with papaya, guava accumulated more zinc but less lead and iron. Using the Hakanson model, the U.S Environmental Protection Agency determined that the daily intake of metal-contaminated guava poses a risk to humans; the estimated health hazard quotients for adults aged 25–55 years and children aged 12–15 years were 1.15 and 1.78, respectively (US EPA 2020). Poor people often think of guava as being similar to apples, but these results prove that it is not healthy to eat in this contaminated areas. The study results are similar to those of^36,37^.

### Metals in coconut water

The presence of lead, cadmium, and zinc in the coconut samples was determined to be due to the metal-contaminated soil in which the coconuts were grown. “It was calculated that Pb (0.004), Zn (0.003), and Cd (0.107) were the concentrations of various metals in the soil and coconut water. Table 10 shows the average concentrations of various metals (mg/kg) in the coconut water samples. The results demonstrated considerable fluctuations in the following order of metal concentrations: the results for Zn, Pb, and Cd were comparable to those for papaya and guava (P < 0.05). These results are consistent with the predicted sequence of metal concentrations in coconut-growing soil. In addition to being free of Al, coconut water was shown to be polluted with Cd.

**Table 10:** Concentrations (mg/L) of different metals in coconut water collected from local area.

Details (mg/L)	Cd (mg/L)	Pb (mg/L)	Zn (mg/L)
Mean	0.01	0.19	0.60
Maximum	0.019	0.41	0.85
Minimum	0.009	0.052	0.36
Std. dev.	0.01	0.11	0.22
Permissible Standard FAO,2020	0.01	0.05	5.00
Higher/lower (fold)	At par	4	(8.33)
Condition	Less nutrient		Less nutrient

Limits of permissible concentration as per FAO/WHO,2020^28,29^.

**Fig. 6:**
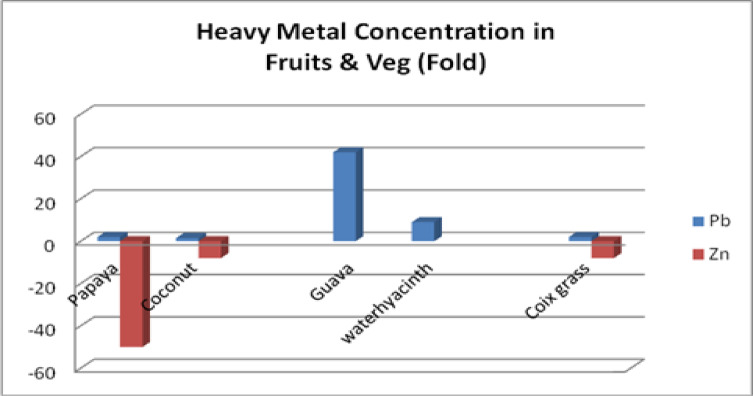
Pb and Zn concentrations in fruits, vegetables, and plants are shown either in higher or lower order (fold), respectively, against WHO/FAO and IS:10500:2020 standards.

Table 10 shows that, according to IS: 10,500 (2020) and WHO (2023), the average amounts of lead (0.19) and cadmium (0.01) in coconut water samples are greater than the allowable limits for drinking water. The metal loadings for Pb and Cd are 3.8- and 3.3-fold greater, respectively, than the FAO/WHO (2020) permitted limits for coconut water, as indicated in Table 10. Further, Fig. 6 shows the heavy metal concentration in different fruit and vegetable products.

In addition to the allowable metal concentration limit as established by the Hakanson model, the heavy metal hazard quotients for adults and children resulting from Pb contamination in coconut water are 3.2 and 2.7, respectively. The amount of zinc in papaya is less than that in guava, which is less than the amount of zinc in coconut, and the amount of lead in papaya is less than that in guava, which is less than the amount of zinc in coconut. Based on these results, coconut water is not potable. These findings are in agreement with those of previous studies by^38–41^. Socioeconomic status, lack of personal cleanliness, and exposure to germs are all risk factors for these illnesses. According to^42^ the impact of each of these elements is not yet clear^43–45^ demonstrated a varying geographic distribution within nations.

This area is experiencing a surge of health difficulties due to the rapid industrialization that has taken place there. The ‘four Ds’—disruption, deprivation, disease, and death—will be used to examine rapid economic expansion. People need to combine new buildings and eco-plan regions to address these problems and their effects. Appropriate regulatory frameworks for preventive health care and social safety nets for the most vulnerable members of society need substantial financial investment in urban regions^46^.

## Health status

According to advanced research, field observations, and surveys among villagers, the local villagers experience gastrointestinal issues, vomiting, and tooth discoloration as a result of consuming tainted produce and water. Digestive issues and hepatitis are examples of significant gastrointestinal (GI) diseases that affect 40% of the population. Table 11 shows the health status as it is according to the surveys and observations.

**Table 11:** Disease profile.

Serial no	Waterborne diseases	Yes (%)	Remarks
1	Digestive problem	78.00%	Related to gastroenterology
2	Diarrhea	12.32%	Related to gastroenterology
3	Dysentery	35.61%	Related to gastroenterology
4	Typhoid	8.00%	–
5	Cholera	7.00%	Related to gastroenterology
6	Hepatitis/liver trouble	6.84%	Related to gastroenterology
7	Vomiting	53.42%	Related to gastroenterology
8	Discoloration of teeth	63.01%	Related to gastroenterology

Other regions of India, as well as China, Turkey, Morocco, France, Italy, Bangladesh, and Pakistan, have all shown similar outcomes. The incidence of pancreatic, bladder, and prostate cancers, in addition to gastrointestinal cancers, has been the subject of prior research^47^. Low, medium, and high quantities of lead, cadmium, and chromium may accumulate in plant shoots and roots^3,48,49^. The study will help next-generation researchers to go ahead with further study on this issue to overcome the situation particularly using metals, and chemicals in process industries, and pharmaceutical/medical industries to manage health situations.

## Socioeconomic conditions of the BD industrial region

A total of 41.09% of those who took part in the poll were locals, whereas 58.91% were only passing through. A sufficient workforce was not available in the region, and some inhabitants were hesitant to conduct this sort of hazardous job, therefore, migrant laborers filled the personnel needs in the cluster. The age categories of 21–40 years and 55 years comprised 78.08% and 17.08% of the respondents, respectively. In terms of education level, 89.04% were literate, 10.96% were illiterate, 45.20% had finished elementary school, and 42.46% had finished secondary school.

According to the study, 56% of the inhabitants work in the textile industry, knitting, printing, or related fields; 32% work in the industries that produce small steel items, grocery stores, wooden furniture, etc.; and 12% work as bus drivers, automobile mechanics, or minibus operators. Table 12 shows that out of the total number of villagers, 1.36% had annual incomes of ≤ Rs.36,000, 86.32% had incomes of ≥ Rs. 60,000 ($870), and 12.32% had incomes of ≥ Rs.1, 20,000 ($1740), in contrast to India’s annual per capita income of Rs.74,380 ($1065) according to MSPI (2015)^50^.

**Table 12:** Socioeconomic profile based on survey.

Sl no.	Particulars	Details	Other information
1	Type of respondent	Male: 84.94%	Female: 15.06%
2	Type of respondent	Local: 41.09%	Migrated:58.91%
3	Age group	21–40 yrs: 78.08%	50–55 year: 17.08%
4	Literacy rate	Literate: 89.04%	Illiterate: 10.96%
5	Type of literacy	Class I—IV: 45.28%	Class V—X:25.00% Class X-XII: 12.00% Higher education: 6.85% Balance NA : 10.95%
6	Type of occupation	Textile bleaching and dyeing: 56.00%	Different small business : 32.00%, automobile and others : 12.00%
7	Annual income	≤Rs. 36,000/- : 1.36%	≥ Rs.60,000/- : 86.32% ≥ 1,20,000/- : 12.32%

## Remedial measure

### Eco-textile park

We must act immediately to relocate the existing units of the large cluster to some planned industrial estates with 200–400 units each. This estate must have a shared effluent treatment facility, which must include pre-treatment, secondary treatment, and membrane-based treatment with water reuse. The area must also undergo eco-planning in-order to acquire water of sufficient quality to lessen the dangers of metal contamination in agro-products, need of textile industries, the negative effects on human health, and the loss of million gallons of underground water resources daily including saving of power and fuel as well.

### Nanoparticle techniques

Nanoparticles (NPs) are currently a fascinating area of research right now, because of their potential to increase soil fertility via agro-nanotechnology and to decrease heavy metal bioavailability^50^. Soil treatment with nano-tools is an economical option. Both normal agricultural development and human health both make use of NPs and environmentally friendly compounds^51^.

**Fig. 7:**
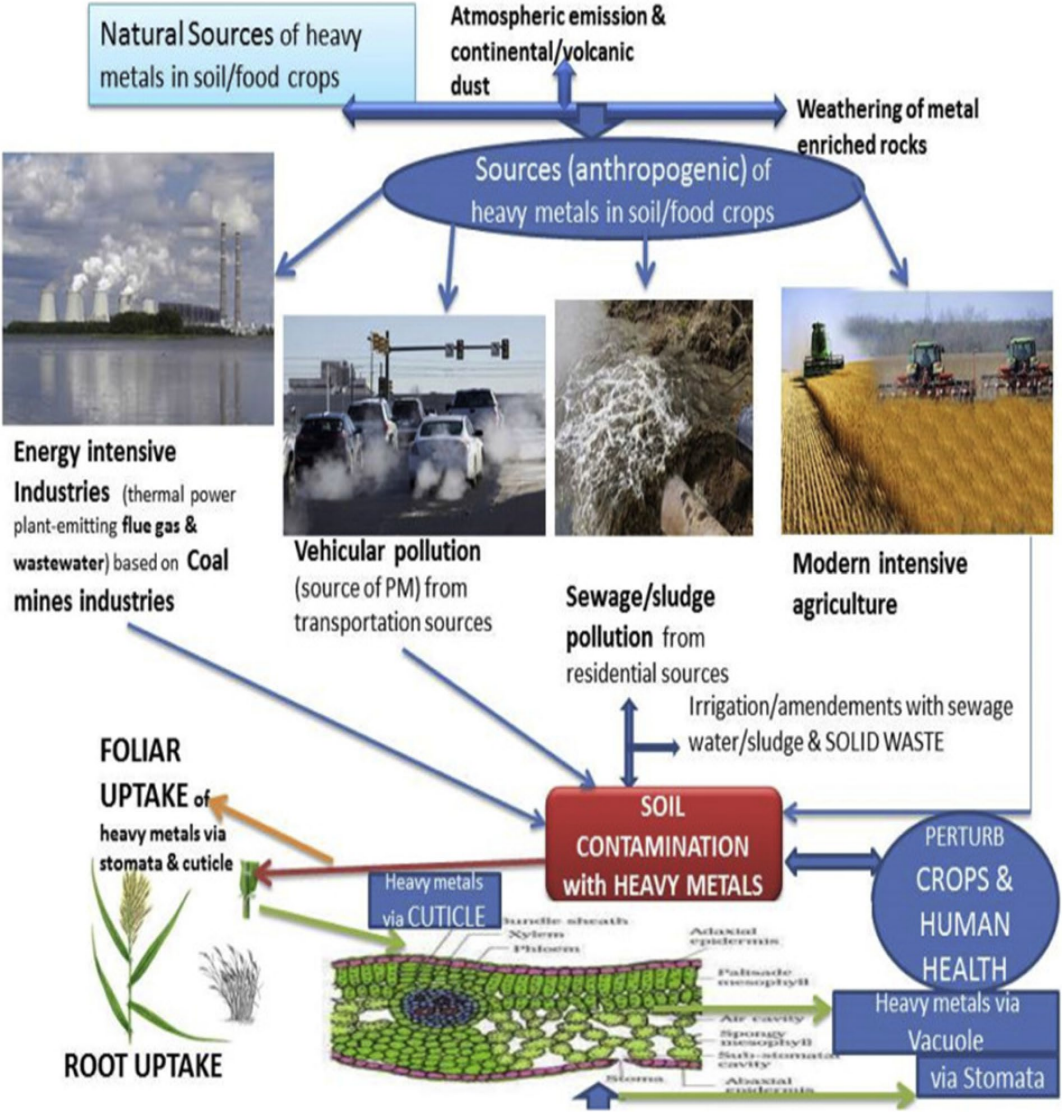
Details of nanoparticle techniques.

Furthermore, nano-sensors have applications in evaluating the safety of food, particularly in determining the levels of contamination in agricultural products^52,53^. As shown in the case of pesticide formulations using different nanotechnologies or formulations, technologies (Fig. 7) are needed to reduce the danger of metal-preamble wastewater and sludge harming food crops^54^. Wheat grown in polluted soil close to factories may have more carcinogenic metals bioavailable after adsorption with charcoal nano-sheets^55^. Silica NPs, on the other hand, increase Cd toxicity in rice by blocking gene activity linked to the synthesis of Cd transporters (OsHMA_3_)^56–58^. Therefore, understanding how NPs affect food crops and the environment, as well as the opposite consequences, is important.

## Principal significance and limitations

This article aims to put together cutting-edge research findings linked with heavy metal effect from wastewater generated by BD textile units on surface water, soil, fruits and vegetable and its resulting effect on public health due to food chain, clubbed with poor socio-economic condition. The disease profile identified gastro-intestinal disorder, diarrhea, liver trouble are the most vulnerable. Aims to save millions of gallons of groundwater, power and energy and to supply drinkable water with the help of membrane-based wastewater treatment plants with recycle and reuse of treated water and eco-planning of the industrial cluster to save thousands of human lives throughout the globe in this age of climate change through restoring ground water condition and unplanned industrial growth.

### Limitation

To restrict unplanned growth of BD units, eco-planning, Wastewater Treatment Facilities.

## Conclusion

Environmental pollutants, food safety and security, and human health are closely interconnected. The levels of heavy metals in the environment have significantly risen in recent decades. The origins of heavy metals in food crops differ between developing and advance countries. The deposition of heavy metals on food crops and the application of industrial effluents are the principal causes of pollution in soil-crop systems in developed nations. In developing nations, irrigation with improperly treated wastewater or its sludge constitutes the primary cause of contamination for food crops. The transport of heavy metals from soil to crop systems is intricate and involves multiple pathways. Multi-metal toxicity in agricultural crops necessitates focused examination to ascertain the precise levels of metal toxicity. Global investigations into human health concerns have been extensive; nevertheless, only a limited number of these studies have employed appropriate epidemiological methodologies. This research indicates that papaya, guava, coconut water, long grass, and water hyacinth had lead (Pb) concentrations exceeding the World Health Organization’s permissible limits. The concentration of cadmium in guava was significantly greater than anticipated. Although the chromium levels in guava were within the typical range, the zinc levels in long grass, papaya, and coconut water were significantly below the FAO/WHO guidelines. Consequently, untreated wastewater infiltrating surface and groundwater, adjacent land, and produce has resulted in elevated heavy metal concentrations in surface water, soil, and fruits and vegetables, surpassing the standards set by WHO/US EPA/IS, thereby posing significant health risks. More than 50% of the population, especially individuals aged 25 to 45 within the industrial sectors, migrants, and rural areas, suffer from gastroenterological disorders, including gastrointestinal ailments, ulcers, and heartburn, as indicated by study. The implementations of public policy as well as participation of cluster’s private association are required to take immediate necessary steps for restoration of the region’s ecosystem, health problems and the recovery of millions gallons of subterranean water resources which may be accomplished through nanoparticle techniques and the eco-planning of industrial zones, including common wastewater treatment facilities with recycling and reuse processes to harmonise industrial operations with ecological sustainability.

## Supplementary Information

Below is the link to the electronic supplementary material.

Supplementary Material 1

## Data Availability

Data is provided within the manuscript or supplementary information files.

## Abbreviations

WHO: World Health Organization
FAO: Food and Agriculture Organization, United Nations
IS: 10500:Indian standard no.10500
APHA: American Public Health Association, USA
