# Supplementary material for: Heavy metal contamination from textile wastewater and its health impacts: a case study from West Bengal with sustainable remediation approaches
Source: Sci Rep. 2025 Aug 12;15:29578. doi: 10.1038/s41598-025-13357-w (PMC12343934; doi:10.1038/s41598-025-13357-w)
Supplement: Supplementary file 1 — Supplementary Material 1 [file 41598_2025_13357_MOESM1_ESM.docx]

**SUPPLEMENTARY FILE**

**Datasets**

**Tables**

**SUPPLEMENTARY** Table 1: Safe Permissible Limit of Heavy Metals in Fruits, Vegetables, Water, and Soil as FAO /WHO standard 2020, and IS: 10500: 2017

| Metal | Fruits/Veg.  ( mg/kg) | Soil  ( mg/kg) | Water  (mg/l) | Papaya  (mg/kg) | Guava  (mg/kg) | Effluent  IS:10500  (mg/l) |
| --- | --- | --- | --- | --- | --- | --- |
| Cu | 73 | 5-5.6 | 2 |  |  | 3.0 |
| Pb | 0.30 | 2-13.4 | 0.01 | Nd | 0.58 | 0.10 |
| Cd | 0.20 | 0.1 | 0.003 | Nd | Nd | 1.0 |
| Cr | 0.1-1 | 10-80 | 0.05 |  |  | 2.0 |
| Zn | 99.4 | 60-780 | 3 |  |  | 15 |
| Ni | 1-10 | 10-50 | 0.02 | 0.26 | Nd | 3 |

Nd= not detectable, Source a=Adue et.al 2012

**SUPPLEMENTARY** Table 2: Pb concentration in effluent, canal, pond, tube well water and soil

| Sl. No | Sample  & year  2022-23 | Canal Water (mg/l) | Effluent (mg/l) | Pond water (mg/l) | Tube well water (mg/l) | Soil  (mg/kg) |
| --- | --- | --- | --- | --- | --- | --- |
| 1. | S-1 | 0.05 | 0.07 | 0.01 | 0.007 | 1.16 |
| 2 | S-2 | 0.05 | 0.08 | 0.25 | ------ | 11.14 |
| 3. | S-3 | 0.104 | ----- | ------ | ------ | 17.32 |
| 4. | S-4 | 0.03 | ----- | ------ | ------ | 41.20 |
| 5. | S- 5 | 0.014 | 0.25 | 0.016 | 0.058 | 90.80 |
| 6. | S-6 | 0.15 | 1.84 | 0.01 | ------ | ------ |
| 7. | S-7 | 0.10 | 0.10 | ------ | ------ | ------ |
| 8. | S-8 | 0.10 | 0.14 | ------ | ------ | ------ |
| 9. | S-9 | ---- | 0.17 | ------ | ------ | ------ |
|  | Mean | 0..38 | 0.074 | 0.07 | 0.02 | 32.32 |
|  | S.D | 0.27 | 0.67 | 0.10 | 0.02 | 32.07 |

S-1-4, S:5-9 indicate samples of the 1st year and 2^nd^ year. As per WHO safe limits of Pb concentration in canal water, Effluent, Pond water, tube well water, and soil were very significantly higher (39-fold, 7-fold, 7-fold, 10-fold). Further, the Mean Pb concentration was found in canal water > soil> effluent ˃ Pond ≥ tube well water. Again, Pb levels increased from the 1^st^ year to the 2^nd^ year in canal water (0.03 mg/l to 0.15 mg/l), effluent (0.07mg/l to 1.84 mg/l), tube well water (0.007 mg/l to 0.058 mg/l), and soil (from 1.16mg/kg to 90.80 mg/

**SUPPLEMENTARY Table 3: Content of Nickel in the effluent, canal water, pond, tube well water, and soil**

| Sample/  Year | Canal water (mg/l) | Effluent  (mg/l) | pond  (mg/l) | Tube well (mg/kg) | Soil(mg/kg) |
| --- | --- | --- | --- | --- | --- |
| S-1 | 0.05 | - | - | - | 0.34 |
| S-2 | 0.05 | - | - | - | 14.93 |
| S-3 | 0.07 | - | - | - | 34.77 |
| S-4 | 0.05 | - | - | - | - |
| S-5 | 0.037 | 0.049 | 0.02 | 0.016 | - |
| S-6 | 0.037 | 0.093 | - | - | - |
| Mean | 0.05 | 0.07 | 0.02 | 0.016 | 16.68 |
| SD | 0.01 | 0.02 | - | - | 14.11 |

---------------------------------------------------------------------------------------------------------------------------------

--------------------------------------------------------------------------------------------------------------------------------

As per the Sample in the 1st Year (S: 1-4) and 2^nd^ year (S: 5-6), Mean Ni in Effluent, canal, pond water, and soil were significantly Higher (4-fold, 3-fold, normal, 2-fold) and lower in Tube well waste

**SUPPLEMENTARY** Table 4: Content of Zinc in the effluent, canal water, pond, tube well water, and soil

----------------------------------------------------------------------------------------------------------

| Sample | Canal water | Effluent | Pond  water | Tube well  water | Soil |
| --- | --- | --- | --- | --- | --- |
|  | Mg/l | Mg/l | Mg/l | Mg/l | Mg/kg |
| S-1 | 0.05 | 0.94 | 0.004 | 1.15 | 250.84 |
| S-2 | 0.28 | 0.15 | ----- | ------ | 54.26 |
| S-3 | 0.02 | ----- | ----- | ------ | 980.4 |
| S-4 | 0.18 | ----- | ----- | ------ | ------- |
| S-5 | 0.15 | 0.55 | 0.05 | 0 | ------ |
| S-6 | ----- | 0.07 | ------ | ------ | ------ |
| S-7 | ----- | 0.09 | ------ | ------ | ------ |
| Mean | 0.14 | 0.36 | 0.027 | 1.15 | 428.50 |
| S.D | 0.09 | 0.33 | 0.025 | ---- | 398.41 |
|  |  |  |  |  |  |

As per WHO limits, the mean Zn level in effluent, canal, pond, tube well water, and soil

Very significantly lower (8- fold, 21-fold, 111- fold, 3- fold, 2- fold).

**SUPPLEMENTARY** Table 5: Content of Cadmium (Cd) in Soil

| Sample/ year | Soil  mg/kg |
| --- | --- |
| S -1 | 0.94 |
| S- 2 | 0.53 |
| R- 3 | 1.29 |
| Mean | 0.92 |
| S.D | 0.31 |

As per WHO standards, the Concentration of Cd in Soil is lower (9 fold)

**SUPPLEMENTARY** Table 6: Metal content in different fruits and vegetables

| \| Metal \| Papaya  mg/kg \| Coconut water  mg/l \| Guava  mg/kg \| Coix grass mg/kg \| Water Hyacinth  mg/kg \| \| --- \| --- \| --- \| --- \| --- \| --- \| \| Pb \| 0.45 \| 0.41 \| 12.62 \| 0.66 \| 2.75 \| \| Cd \| ---- \| ---- \| 1.47 \| 0.10 \| 0.15 \| \| Cr \| ---- \| ---- \| 0.10 \| 0.145 \| 0.27 \| \| Zn \| 1.96 \| 0.39 \| ---- \| 13.15 \|  \| \|  \|  \|  \|  \|  \|  \| \|  \|  \|  \|  \|  \|  \| \|  \|  \|  \|  \|  \|  \| \|  \|  \|  \|  \|  \|  \|   Pb levels are significantly higher (2-fold, 1.5-fold, 42-fold, 2-fold, 9-fold), safe limits of Cd in  Guava were significantly higher (7-fold), the Safe limit of Cr in Guava was normal, and the Safe  limit of Zn in Papaya, Coconut water, and Coix grass were significantly Lower, 50-fold, 8–fold,  and 8 fold, respectively.  **SUPPLEMENTARY** Table 7: Concentrations of different metals in green papaya  ------------------------------------------------------------------------------------   \| Parameters \| Na \| Pb \| Fe \| Zn \| Al \| \| --- \| --- \| --- \| --- \| --- \| --- \| \| Samples \| mg/kg \| mg/kg \| mg/kg \| mg/kg \| mg/kg \| \| S1 \| 65.88 \| 0.51 \| 7.06 \| 1.64 \| 5.1 \| \| S2 \| 66.72 \| 0.62 \| 10.86 \| 3.01 \| 7.56 \| \| S3 \| 64.13 \| 0.27 \| 9.75 \| 2.46 \| 3.85 \| \| S4 \| 72.91 \| 0.69 \| 6.97 \| 3.01 \| 8.13 \| \| S5 \| 65.86 \| 0.3 \| 7.59 \| 1.66 \| 7.84 \| \| S6 \| 63.46 \| 0.75 \| 11.57 \| 2.2 \| 3.88 \| \| S7 \| 62.11 \| 0.45 \| 3.25 \| 1.96 \| 7.56 \| \| **Mean** \| **65.87** \| **0.51** \| **8.15** \| **2.28** \| **6.27** \| \| Maximum \| 72.91 \| 0.75 \| 11.57 \| 3.01 \| 8.13 \| \| Minimum \| 63.46 \| 0.27 \| 3.25 \| 1.64 \| 3.85 \| \| Std.Dev \| 3.49 \| 0.19 \| 2.84 \| 0.58 \| 1.92 \| \| FAO/WHO  (2020) \| --- \| 0.10 \| 5.0 \| 3.0 \| 6.00 \| \| Condition/  Fold higher \| --- \| 5 \| 1.6 \| low \| 1.05 \|   **-----------------------------------------------------------------------**    **SUPPLEMENTARY** Table 8: Health risk (HRI) assessment due to metals in Papaya   \|  \| Unit  mg/kg \| HQ Adult \| HQ Child \| DDI \| DIM \| HRI \| \| --- \| --- \| --- \| --- \| --- \| --- \| --- \| \| Mean Pb concentration \| 0.51 \| 8.70 \| 7.28 \| 0.0008 \| 0. 003 \| 0.86 \| \| Mean Al concentration \| 5.1 \| 4.60 \| 3.21 \| 0.0001 \| 0.001 \| 0.21 \| \| Total HRI \|  \|  \|  \|  \|  \| 1.07 \| \| HQ Standard \|  \| 1.00 \| 1.00 \|  \|  \| 1.00 \|   DDI=Daily dietary Index, DIM=Daily intake of metal , HRI= Health risk index, Adult=55 year  Based on US EPA(2020) and Hakanson (1980) for HQ and US EPA (2020) for HRI  **SUPPLEMENTARY Table 9: Concentrations (mg/L) of different metals in Guava**   \| Samples \| Unit \| Cd \| Pb \| Zn \| \| --- \| --- \| --- \| --- \| --- \| \| S1 \| mg/L \| 0.005 \| 0.41 \| 0.36 \| \| S2 \| mg/L \| 0.017 \| 0.052 \| 0.39 \| \| S3 \| mg/L \| 0.009 \| 0.161 \| 0.775 \| \| S4 \| mg/L \| 0.019 \| 0.172 \| 0.835 \| \| S5 \| mg/L \| 0.009 \| 0.181 \| 0.431 \| \| S6 \| mg/L \| 0.019 \| 0.18 \| 0.853 \| \| S7 \| mg/L \| 0.009 \| 0.197 \| 0.566 \| \| Mean \|  \| 0.01 \| 0.19 \| 0.6 \|  \| Maximum \| \| 0.02 \| 0.41 \| 0.85 \| \| --- \| --- \| --- \| --- \| --- \| \|  \| \|  \|  \|  \| \| Minimum \| \| 0.01 \| 0.52 \| 0.85 \| \| Std. dev. \|  \| 0.01 \| 0.11 \| 0.22 \| \| FAO/WHO  (2020) \|  \| 0.003 \| 0.01 \| 5.00 \|   **SUPPLEMENTARY Table 10: Concentrations (mg/L) of different metals in coconut**  **water collected from local area**   \| Details (mg/L) \| Cd(mg/L) \| Pb(mg/L) \| Zn(mg/L) \| \| --- \| --- \| --- \| --- \| \| Mean \| 0.01 \| 0.19 \| 0.60 \| \| Maximum \| 0.019 \| 0.41 \| 0.85 \| \| Minimum \| 0.009 \| 0.052 \| 0.36 \| \| Std. Dev. \| 0.01 \| 0.11 \| 0.22 \| \| Permissible  Standard FAO,2020 \| 0.01 \| 0.05 \| 5.00 \| \| Higher/Lower(Fold) \| At par \| 4 \| (8.33) \| \| Condition \| Less nutrient \|  \| Less nutrient \|   Note: limits of permissible concentration as per FAO/WHO,2020  **SUPPLEMENTARY** T**able 11: Disease profile**   \| Serial no \| Waterborne diseases \| Yes (%) \| Remarks \| \| --- \| --- \| --- \| --- \| \| 1 \| Digestive problem \| 78.00% \| Related to Gastroenterology \| \| 2 \| Diarrhea \| 12.32% \| Related to Gastroenterology \| \| 3 \| Dysentery \| 35.61% \| Related to Gastroenterology \| \| 4 \| Typhoid \| 8.00% \| ----- \| \| 5 \| Cholera \| 7.00% \| Related to Gastroenterology \| \| 6 \| Hepatitis/liver trouble \| 6.84% \| Related to Gastroenterology \| \| 7 \| Vomiting \| 53.42% \| Related to Gastroenterology \| \| 8 \| Discoloration of teeth \| 63.01% \| Related to Gastroenterology \|   **SUPPLEMENTARY Table 12: Socioeconomic profile based on survey**   \| Sl No. \| Particulars \| Details \| Other information \| \| --- \| --- \| --- \| --- \| \| 1 \| Type of respondent \| Male: 84.94% \| Female: 15.06% \| \| 2 \| Type of Respondent \| Local: 41.09% \| Migrated:58.91 % \| \| 3 \| Age group \| 21—40yrs: 78.08% \| 50 -55 yr: 17.08% \| \| 4 \| Literacy rate \| Literate: 89.04% \| Illiterate: 10.96% \| \| 5 \| Type of Literacy \| Class I—IV: 45.28% \| Class V—X:25.00 %  Class X-XII: 12.00%  Higher Education: 6.85 %  Balance NA : 10.95% \| \| 6 \| Type of Occupation \| Textile Bleaching and dyeing: 56.00 % \| Different small business : 32.00%, Automobile & 0thers : 12.00% \| \| 7 \| Annual Income \| ≤Rs. 36,000/- : 1.36 % \| ≥ Rs.60,000/- : 86.32%  ≥1,20,000/- : 12.32% \| |
| --- | --- | --- | --- | --- | --- | --- | --- | --- | --- | --- | --- | --- | --- | --- | --- | --- | --- | --- | --- | --- | --- | --- | --- | --- | --- | --- | --- | --- | --- | --- | --- | --- | --- | --- | --- | --- | --- | --- | --- | --- | --- | --- | --- | --- | --- | --- | --- | --- | --- | --- | --- | --- | --- | --- | --- | --- | --- | --- | --- | --- | --- | --- | --- | --- | --- | --- | --- | --- | --- | --- | --- | --- | --- | --- | --- | --- | --- | --- | --- | --- | --- | --- | --- | --- | --- | --- | --- | --- | --- | --- | --- | --- | --- | --- | --- | --- | --- | --- | --- | --- | --- | --- | --- | --- | --- | --- | --- | --- | --- | --- | --- | --- | --- | --- | --- | --- | --- | --- | --- | --- | --- | --- | --- | --- | --- | --- | --- | --- | --- | --- | --- | --- | --- | --- | --- | --- | --- | --- | --- | --- | --- | --- | --- | --- | --- | --- | --- | --- | --- | --- | --- | --- | --- | --- | --- | --- | --- | --- | --- | --- | --- | --- | --- | --- | --- | --- | --- | --- | --- | --- | --- | --- | --- | --- | --- | --- | --- | --- | --- | --- | --- | --- | --- | --- | --- | --- | --- | --- | --- | --- | --- | --- | --- | --- | --- | --- | --- | --- | --- | --- | --- | --- | --- | --- | --- | --- | --- | --- | --- | --- | --- | --- | --- | --- | --- | --- | --- | --- | --- | --- | --- | --- | --- | --- | --- | --- | --- | --- | --- | --- | --- | --- | --- | --- | --- | --- | --- | --- | --- | --- | --- | --- | --- | --- | --- | --- | --- | --- | --- | --- | --- | --- | --- | --- | --- | --- | --- | --- | --- | --- | --- | --- | --- | --- | --- | --- | --- | --- | --- | --- | --- | --- | --- | --- | --- | --- | --- | --- | --- | --- | --- | --- | --- | --- | --- | --- | --- | --- | --- | --- | --- | --- | --- | --- | --- | --- | --- | --- | --- | --- | --- | --- | --- | --- | --- | --- | --- | --- | --- | --- | --- | --- | --- | --- | --- | --- | --- | --- | --- | --- | --- | --- | --- | --- | --- | --- | --- | --- | --- | --- | --- | --- | --- | --- | --- | --- | --- | --- | --- | --- | --- | --- | --- | --- | --- | --- | --- | --- | --- |

**SUPPLEMENTARY Graphical Abstract**

**
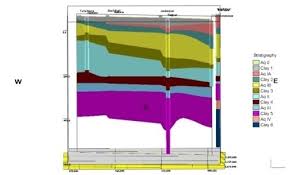
**

**SUPPLEMENTARY** Fig 1: Groundwater map in South 24 Parganas, West Bengal


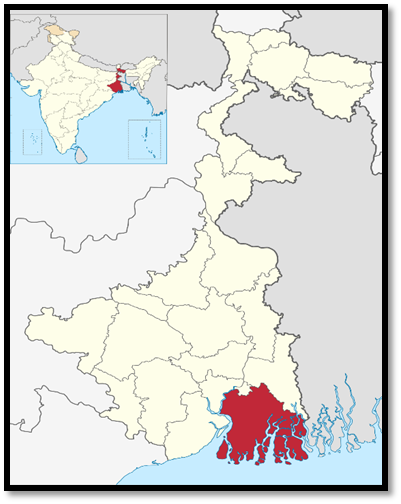


**SUPPLEMENTARY** Fig .2 Maps of South 24 Parganas, West (source Wikimedia commons) Free to use,


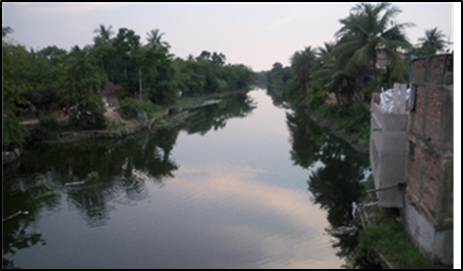


Supplementary Fig.3 Map of Contaminated Sarface water.

of South 24 parganas., &West Bengal


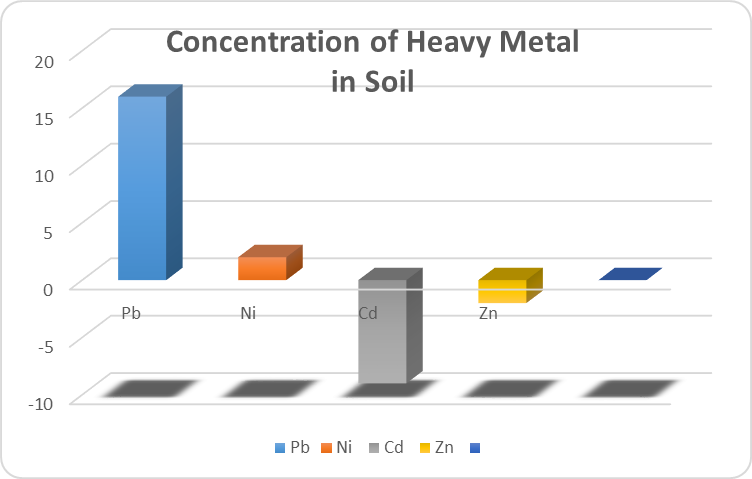


Supplementary Figure 4:. The concentration of different heavy metals in soils collected from agricultural land in the vicinity of the bleaching and dyeing units. Respective concentrations have been represented in proportionate to increased or decreased levels expressed in folds.


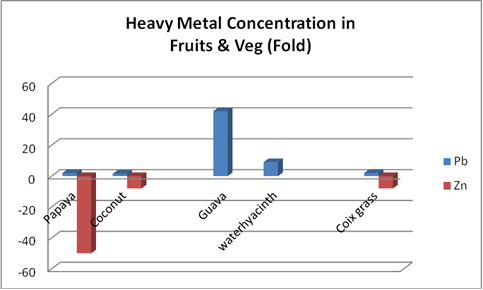


Supplementary Figure 5 : Pb and Zn concentrations in fruits, vegetables, and plants are shown either in higher or lower order (fold), respectively, against WHO/FAO and IS:10500:2020 standards.
